# A case–control study on health-related quality of life of systemic lupus erythematosus patients

**DOI:** 10.1038/s41598-024-55833-9

**Published:** 2024-03-04

**Authors:** Rania H. Refai, Mohammed F. Hussein, Mamdouh H. Abdou, Anna N. Abou-Raya

**Affiliations:** 1 Department of Medicine Supply and Pharmacy, Alexandria University Hospitals, Alexandria University, Alexandria, Egypt; 2 High Institute of Public Health, Alexandria University, Alexandria, Egypt; 3 Faculty of Medicine, Alexandria University, Alexandria, Egypt

**Keywords:** SLE, Health-related quality of life, SF-36, SLE burden, Immunology, Diseases, Rheumatology

## Abstract

Systemic lupus erythematosus (SLE) is a chronic rheumatic autoimmune condition that can cause a wide range of symptoms and problems that may affect the health-related quality of life. The main objective of the study was to assess the SLE burden by exploring the effect of the disease on health-related quality of life. The study consisted of 29 female SLE patients and 27 healthy female controls; they were matched for age and parity. A 36-item Short Form health survey questionnaire (SF-36) was used to collect data from participants through face-to-face interviews and to assess their health-related quality of life. SF-36 summary scores for the physical and mental components were decreased in the studied patients compared with controls; PCS was 28.81 ± 16.63, 77.25 ± 15.75 for cases and controls, respectively; and MCS was 32.75 ± 18.69, and 78.75 ± 10.63 for cases and controls, respectively (p < 0.05). The high correlation between the two dimensions characterizes this decrease. SLE negatively affected the quality of life of the patients. Measures such as lifestyle modifications, physical activity, and a healthy diet should be taken to improve the health-related quality of life in SLE patients. In addition, raising the patient’s awareness about the disease and its consequences could help to cope with the illness and engage in social and physical activities.

## Introduction

Systemic lupus erythematosus (SLE) is a rheumatic autoimmune condition with a considerable individual and public health impact. It could be manifested by a large variety of symptoms and problems^1^. It affects mostly women, especially those of childbearing age^2^. It is a multi-system disease that can affect almost any organ, including the brain, skin, muscles, joints, kidneys, heart, lungs, blood, and also the central nervous system, resulting in serious organ complications and even death^1^.

SLE patients frequently present with non-specific symptoms, such as fatigue, arthralgia, and general malaise. Patients’ symptoms vary and may be mild or severe. Patients may experience immunological and neurological diseases, renal difficulties, rash, arthritis, photosensitivity after exposure to ultraviolet (UV) radiation, and more^3^.

Nearly everyone with SLE experiences fatigue, which can be so incapacitating that participation in social gatherings and/or daily activities may be impossible. It may have an impact on every facet of a patient's quality of life and have an impact on their physical and mental health^4^. In a European survey, 85.3% of patients pinpointed fatigue as their most incapacitating symptom^5^.

Aspects of life that are impacted by health, such as functional, social, or emotional status, are referred to as health-related quality of life (HRQoL)^6^. Three domains, including reversible disease activity, irreversible accumulated damage, and health-related quality of life, are used to describe the burden of SLE. It was discovered that the severity of the disease and the incidence of organ damage both had a substantial impact on the HRQoL of SLE patients^7^.

The chronic nature of SLE has also been observed to affect patients’ HRQoL; a Swedish study indicated that SLE patients reported low HRQoL and high medical costs that correlate with disease activity^8^. Moreover, an Italian study concluded that SLE patients had lower physical and mental scores, contributing to the decrease in HRQoL compared with controls^9^.

The most frequently used valid generic Patient-Reported Outcomes (PRO) instrument to assess how an illness affects HRQoL is the 36-item Short Form Health Survey questionnaire (SF-36). It is useful for comparing the health status of patients to that of the general population. It was recommended as the instrument of choice for assessing HRQoL in SLE^10–12^.

The current study assessed the impact of the disease on HRQoL in Egypt in order to enable better insight into the patients’ needs and to attract the attention of physicians to this missed part of the management plan to provide better support to SLE patients.

## Patients and methods

### Study setting and design

This case–control study was conducted at the rheumatology outpatient clinic of Alexandria Main University Hospital. The study was conducted during the period from June 15, 2020, to September 15, 2020. There were 56 adults in the sample, 29 cases, and 27 controls.

### Study participants

The cases were female patients with idiopathic SLE according to the systemic lupus international Collaborating Clinics (SLICC) criteria^13^. Healthy female volunteers who accompanied the patients to the rheumatology outpatient clinic served as the controls. The patients were previously diagnosed with SLE in the past (within 6 months to 1 year) by a rheumatologist based on their recognition of distinctive symptoms and signs in the context of supporting serologic investigations using the 2012 SLICC criteria, after ruling out other possible diagnoses.

They attended the rheumatology outpatient clinic to follow up during a flare or relapse. The diagnosis was confirmed by the 2012 SLICC criteria. The diagnostic sensitivity, specificity and accuracy of 2012 SLICC criteria were confirmed in a recent Scandinavian study^14^. The rheumatologist helped to choose the cases based on the inclusion and exclusion criteria. Female patients with idiopathic SLE were our inclusion criteria. Exclusion criteria included participants with any other rheumatic diseases, coexisting morbidity unrelated to SLE, such as diabetes or hypertension, cancer, dementia, or psychosis or overlap syndrome to avoid confounding factors. Also, recently diagnosed SLE patients and those diagnosed 3 years ago or more were excluded. Controls were also confirmed by physician examination.

### Sampling design

A total of 29 cases and 27 controls were included in the study. After meeting the inclusion criteria, cases were chosen by a simple random sampling technique, and controls were matched to cases in terms of age, sex and parity.

### Data collection methods

All participants (cases and controls) completed a structured interview questionnaire (15–20 min) that had been pre-designed and pre-coded. Data collection from all participants was done using the Arabic version. There were two sections in the questionnaire. Age, marital status, and employment-related items were included in the first section of the questionnaire.

The standardized and reliable 36-item Short Form Health Survey questionnaire (SF-36) was included in the second section. It was used to gather information about the effect of SLE on health-related quality of life through 36 questions (9–44). This questionnaire evaluates eight of the most significant health concepts, which represent basic human values that are crucial to everyone’s functional status and well-being. Each domain has a variety of items, for example, the physical functioning domain (PF), which measures how well one performs physical activity (10 items); role limitations in usual role activities such as work and everyday tasks because of physical health problems (RP) (4 items); role limitations in usual role activities because of emotional problems (RE) (3 items); bodily pain (BP) which is a two-item scale that measures the limitations due to pain; General mental health (MH) that measures the psychological status and the different feelings such as nervousness, depression, calmness, and happiness (5 items); vitality (VIT) that assesses respondents’ levels of exhaustion and lack of energy (4 items); social functioning (SF) that detects how social activities are affected by physical and emotional health (2 items); and general health perceptions (GH) which assesses five different aspects of personal health. In addition to a single-item measure of health transition that is also included in the SF-36 survey, it is not used to score any of the eight multi-item scales (question 10).

The physical and mental health components of the eight main domains can be combined into two main summary measures. The physical health component score (PCS), is made up of the PF, RP, BP, and GH, each of which measures a different aspect of physical health; PF measures limitations in behavioral performance of everyday physical activities; RP measures the extent of disability in everyday activities due to physical problems; BP focuses on the severity of pain and the resulting limitations in activities.

The second main summary measure is the mental health component score (MCS), which is made up of MH, RE, SF, and VIT. MH focuses on determining whether a person’s mental state—such as their level of anxiety or happiness—was impacted; RE measures the extent of disability in everyday activities due to emotional problems (such as feeling depressed or anxious); SF assesses a person's capacity to engage in the normal way of life in society. The English version of the questionnaire is in the supplementary file^15^.

In order to enable patients with various educational backgrounds to respond to all questions without linguistic restrictions, the valid and reliable Arabic version of the form was used^16^. The Likert method of summarizing responses was used to build the SF-36 items for scoring, with the lowest response receiving a score of 1^17^.

The item scores were added to determine the raw score for each of the eight SF-36 dimensions, which was then translated into a value for the dimension ranging from 0 (poor health, and greater disability) to 100 (better health, and less impairment)^18,19^.

$$Tranformed\;scale = \frac{Actual\;raw\;score - Lowest\;possible\;score}{{Possible\;raw\;score\;range}} \times 100.$$

The study tested the differences in each domain between cases and controls, as well as the relations of the different domains with each other.

### Ethics approval and consent to participate

The study was approved by the High Institute of Public Health’s Ethics Committee in Alexandria, Egypt, in 2019. The study was permitted to be conducted at the Rheumatology Outpatient Clinics of Alexandria Main University Hospital after submitting a formal written request. The Declaration of Helsinki’s ethical guidelines were followed during the study execution. All study participants signed an informed consent form after being told of the goals and advantages of the investigation. Both anonymity and confidentiality were ensured. All procedures were carried out following the applicable rules and regulations.

### Statistical design

The researcher used the Statistical Package for Social Sciences (SPSS) version 21 for data entry and analysis. When the significance probability was less than 5% (p < 0.05), a result was deemed statistically significant. The number and percentage of cases were used to characterize the qualitative variables. Tests of normality were done for quantitative continuous variables. If the variable has a normal distribution, mean and standard deviation (mean ± SD) were computed; otherwise, the median and interquartile range (median ± IQR) were used. The continuous, non-normally distributed variables were subjected to the non-parametric Mann–Whitney–Wilcoxon test (U), to look for differences between the means of the two groups. Spearman’s rank correlation was used to test the association between quantitative variables and to describe the relationship between them.

### Institutional review board statement

This study was approved by the High Institute of Public Health, Alexandria University, Alexandria, Egypt.

## Results

In the present study, Cases were represented during flare most commonly by malar rash, discoid rash, ulcers, arthritis, photosensitivity, alopecia, renal complications, hematologic disorders (including anemia, leukopenia, thrombocytopenia and lymphopenia), immunological disorders including production of autoantibodies, and neurologic disorders including seizures.

Table 1 displays the socio-demographic characteristics of the studied sample. The age ranged from 21 to 59 years. The mean age for cases was 37.48 ± 8.38, and the mean age for controls was 37.81 ± 9.87. About two-thirds of the cases (69%) were aged 35 or more (middle-aged population in the third decade). Most of the participants were married (93.2% for cases and 85.2% for controls). Around two-thirds of the patients received a low level of education, compared to about 60% of the control group, who had a higher level of education.

**Table 1:** Distribution of the studied sample regarding some socio-demographic characteristics and estimation of the risk of SLE in relation to them (Rheumatology outpatient clinic, Alexandria University Hospital, Alexandria, 2020).

Some socio-demographic characteristics	Cases (n = 29)	Controls (n = 27)
	N (%)	N (%)
Age		
18–	2 (6.9)	2 (7.4)
25–	7 (24.1)	10 (37.0)
35+	20 (69.0)	15 (55.6)
Mean ± SD	37.48 ± 8.378	37.81 ± 9.872
Marital status		
Married	27 (93.2)	23 (85.2)
Single	1 (3.4)	3 (11.1)
Divorced/widow	1 (3.4)	1 (3.7)
Crowding index		
< 1	2 (6.9)	3 (11.1)
1–	12 (41.4)	13 (48.2)
2+	15 (51.7)	11 (40.7)
Educational level		
Illiterate/primary	20 (68.9)	7 (25.9)
Secondary	8 (27.7)	16 (59.3)
University/post graduated	1 (3.4)	4 (14.8)
Work		
Not working	25 (86.2)	9 (33.3)
Working	4 (13.8)	18 (66.7)

By evaluating the crowding index, a measure of socioeconomic status that is calculated by dividing the family size by the total number of rooms in the house, excluding the kitchen and bathroom, it was found that 51.7% of cases fell into the crowding index category of two or more compared to 40.7% of controls, indicating that more cases than controls reside in homes that are overcrowded. In terms of occupation, housewives accounted for the bulk of cases (86.2%) as opposed to controls (33.3%).

Data presented in Table 2 were derived from the SF-36 survey responses and the calculation of domain and summary scores for each individual. The results of the study showed great variability in all the subscales between cases and controls. All SF-36 scores showed a remarkable decrease in the case group; all health domains were impacted by HRQoL, and patients responded that they have limitations due to their suffering from SLE.

**Table 2:** Differences in summary and domain mean scores of the SF-36 for HRQoL between SLE cases and related controls (Rheumatology outpatient clinic, Alexandria University Hospital, Alexandria, 2020).

Scale		Cases	Controls	U (p-value)
PF	Median ± IQR	45 ± 30	85 ± 40	88.5*
	Mean rank	18.05	39.72	
RP	Median ± IQR	0	100	0.5*
	Mean rank	15.02	42.98	
RE	Median ± IQR	0	100	15*
	Mean rank	15.52	42.44	
BP	Median ± IQR	41 ± 19	74 ± 38	63.5*
	Mean rank	17.19	40.65	
VIT	Median ± IQR	45 ± 25	70 ± 20	66*
	Mean rank	17.28	40.56	
SF	Median ± IQR	50 ± 31.25	87.5 ± 25	77*
	Mean rank	17.66	40.15	
GH	Median ± IQR	25 ± 17.5	62 ± 10	28.5*
	Mean rank	15.98	41.94	
MH	Median ± IQR	44 ± 26	64 ± 16	91.50*
	Mean rank	18.16	39.61	
PCS	Median ± IQR	28.81 ± 16.63	77.25 ± 15.75	11*
	Mean rank	15.38	42.59	
MCS	Median ± IQR	32.75 ± 18.69	78.75 ± 10.63	40*
	Mean rank	16.38	41.52	

*IQR* interquartile range, *U* Mann–Whitney non-parametric test, *PF* physical functioning, *RP* role physical, *RE* role emotional, *BP* bodily pain, *VIT* vitality, *SF* social functioning, *GH* general health, *MH* mental health, *PCS* physical component score, *MCS* mental component score.

*p < 0.001.

SLE patients have significantly lower physical and mental scores than controls in all SF-36 domains, including general health, physical functioning, physical limitations, emotional limitations, energy/fatigue, emotional well-being, pain, social functioning, and health changes (p < 0.05%). In addition, SF-36 summary scores for the physical and mental components were decreased in the studied patients compared with controls (median ± IQR of PCS = 28.81 ± 16.63, 77.25 ± 15.75 for cases and controls, respectively, and MCS was 32.75 ± 18.69, and 78.75 ± 10.63 for cases and controls, respectively).

The major differences between cases and controls were observed in RP and RE, wherein in the RP domain the lowest sum of raw item scores was 4 and the highest sum of raw item scores was 8, leading to a percent value of 0 and 100 for cases and controls, respectively when calculating the transformed scale. The same appears with the RE domain, where the lowest score was 3 and the highest one was 6, leading to a percent value of 0 and 100 for cases and controls, respectively as illustrated in Table 3. In the cases group, PCS mean scores were found to be lower than MCS mean scores (26.18 ± 9.94 vs. 32.49 ± 12.45).

**Table 3:** Formulas for scoring and transforming scales.

Scale	Sum of the question values “Question number”	Lowest and highest possible raw scores	Possible raw score range
Physical functioning	11 + 12 + 13 + 14 + 15 + 16 + 17 + 18 + 19 + 20	10, 30	20
Role-physical	21 + 22 + 23 + 24	4, 8	4
Bodily pain	29 + 30	2, 12	10
General health	9 + 41 + 42 + 43 + 44	5, 25	20
Vitality	31 + 35 + 37 + 39	4, 24	20
Social functioning	28 + 39	2, 10	8
Role-emotional	25 + 26 + 27	3, 6	3
Mental health	32 + 33 + 34 + 36 + 38	5, 30	25

A highly significant correlation was observed between most of the different domains in the cases group as presented in Table 4, such as between the mental and physical component scores, PF domain and BP, PF domain and VIT domain, RP domain and RE, BP and VIT, and BP with the SF domain, as well as VIT with the SF domain.

**Table 4:** Spearman’s rank correlation (ρ) between the different domains of SF-36 for cases (N = 29) (Rheumatology outpatient clinic, Alexandria University Hospital, Alexandria, 2020).

	PF	RP	RE	BP	VIT	SF	GH	PCS	MCS
PF	1.000	0.118	− 0.027	0.777**	0.727**	0.722**	0.496**	0.915**	0.633**
RP	0.118	1.000	0.812**	0.232	0.291	0.265	− 0.188	0.265	0.492**
RE	− 0.027	0.812**	1.000	0.197	0.167	0.302	− 0.216	0.103	0.503**
BP	0.777**	0.232	0.197	1.000	0.828**	0.863**	0.514**	0.847**	0.836**
VIT	0.727**	0.291	0.167	0.828**	1.000	0.729**	0.536**	0.806**	0.832**
SF	0.722**	0.265	0.302	0.863**	0.729**	1.000	0.506**	0.807**	0.890**
GH	0.496**	− 0.188	− 0.216	0.514**	0.536**	0.506**	1.000	0.686**	0.434*
PCS	0.915**	0.265	0.103	0.847**	0.806**	0.807**	0.686**	1.000	0.764**
MCS	0.633**	0.492**	0.503**	0.836**	0.832**	0.890**	0.434*	0.764**	1.000

*PF* physical functioning, *RP* role physical, *RE* role emotional, *BP* bodily pain, *VIT* vitality, *SF* social functioning, *GH* general health, *MH* mental health, *PCS* physical component score, *MCS* mental component score.

**Correlation is significant at the 0.01 level (2-tailed).

*Correlation is significant at the 0.05 level (2-tailed).

Fatigue affected the other domains of the SF-36 questionnaire, which means that the feeling of tiredness and loss of energy affected the quality of life of the SLE patients. The VIT domain that measured the degree of fatigue in SLE patients had a significantly strong positive correlation with PCS (ρ = 0.806, p = 0.01) and MCS (ρ = 0.832, p = 0.01). To summarize, there is a large SLE burden on HRQoL, which was significantly reduced in SLE patients. The high correlation between the two dimensions characterizes this decrease.

## Discussion

SLE patients experience a wide range of medical and psychological problems, which may have an impact on their quality of life in terms of physical, social, and psychological aspects as well as employment^20^. Since obtaining an updated view of HRQoL is pivotal to our understanding of the disease burden, this study is the first in the Alexandria Governorate to assess HRQoL in SLE patients.

A high crowding index was observed in the SLE patients in the current study. Similar to this study, Pons-Estel et al., in a Latin American multiethnic cohort study, stated that a high crowding index for patients with SLE led to more severe consequences of the disease, which reflected on the daily activities of the patients^21^.

Regarding occupation, other studies found similar results to our research, as the majority of SLE patients were unemployed^22,23^. A high rate of unemployment (59%) was also found in a large group of Dutch SLE patients (N = 147), which was reported due to job loss as a result of disease functional limitations and reduced quality of life^24^.

A kind of limitation from various activities was observed; patients consequently encountered a variety of physical, psychological, and social issues. This could be due to several factors, such as the disease course as an autoimmune disease affecting body organs, the severity of the illness, as well as the disease progression that could be due to a lack of adherence to treatment, absence of regular physician visits, and regular laboratory examinations to detect the disease progression.

Furthermore, the side effects of medication can also have an impact on his well-being. The main drugs that were prescribed for those patients were corticosteroids or immunosuppressive drugs. They have a beneficial effect on the remission of the disease, hence improving the quality of life. However, the misuse of these medications could lead to many side effects that worsen the quality of life. Patients from low socioeconomic classes have low awareness about the benefits and costs of these drugs, and the medical staff have limited time to discuss this issue with the patients. So, the way in which the medications are consumed could affect the quality of life of the patients, which will be reflected in the results. A recent cross-sectional study concluded that medication compliance should be one of the main focuses for a better HRQoL in SLE patients^25^.

Regarding the impact of SLE on HRQoL, the significantly low scores of the SF-36 in the cases in the current study show a bad quality of life, which concurs with those reported by others. Rinaldi et al. found the same observations. Italian SLE patients had lower physical (PCS) and mental (MCS) component summary scores as compared to controls^9^. Several studies confirmed this observation and concluded that the QoL of SLE patients was found to be impaired compared with controls, irrespective of the instrument used in HRQoL assessment^26–28^.

In an Egyptian study conducted at Tanta University Hospital, all domains were found to be lower in SLE patients, and impairment of physical and emotional domains of QoL with disease activity was also observed^29^. Using the Lupus QoL questionnaire to assess QoL, Jolly et al. found that HRQoL was adversely affected, and that longer disease duration was associated with lower QoL scores in SLE patients^30^. Using the same instrument to assess QoL, an Egyptian study conducted at Zagazig University Hospitals concluded that SLE was associated with a considerable burden to the patient^31^.

The low SF-36 scores in SLE patients could reflect the dramatic reduction or even discontinuation of the routine activities that were considered as an important part of their everyday lives, such as school, work, or social relationships. It was also noticed that in the case group, PCS mean scores were lower than MCS mean scores (26.18 ± 9.94 vs. 32.49 ± 12.45) which suggested that physical health was more impaired than mental health in SLE patients in the present study.

The highly significant correlation between most of the domains in the cases group could explain that most of the aspects were related to each other and the defect in one domain could be reflected in other domains. So, the different aspects of quality of life are interrelated.

In our study, the VIT domain that measured the degree of fatigue in SLE patients had a significant strong positive correlation with PCS (ρ = 0.806, p = 0.01) and MCS (ρ = 0.832, p = 0.01). This supports a study that suggested the importance of intervention strategies that target anxiety and depression to alleviate fatigue^32^.

## Conclusion and recommendations

According to the results of the current case–control study, it was determined that SLE had a considerable negative impact on the health-related quality of life (HRQoL) of patients and their ability to carry out normal daily activities, resulting in a high prevalence of disability. The HRQoL of patients with SLE was consistently lower than that of matched healthy controls. Both the physical and mental aspects of HRQOL are much lower in SLE patients, and this decline is characterized by a strong correlation between the two categories.

HRQoL in SLE should be a priority objective, as patients not only aspire to live longer but also to live better and practice their daily routine normally. In addition, increasing the patients’ awareness about the disease and its consequences is necessary to help them cope with the illness and prevent any exposures that would increase the risk. More large-scale research may be crucial to provide more representation of patients with SLE in Egypt and to find more effective treatment plans and strategies to increase the quality of life for lupus patients.

## Limitations and strengths

The first limitation of the study was the small sample size. Due to the emerging coronavirus disease 2019 (COVID-19) during the period of the study, there was limited access to the patients. Second, the effect of social isolation on the quality of life in the COVID-19 era as a confounder was not studied. However, in this early period of the pandemic, Egypt did not implement strict social isolation precautions. Third, the inherent limitations of the adopted study design (case–control), such as recall bias and inability to assess causality.

Our study had many points of strength. To the best of our knowledge, this is the first study that addressed HRQoL among patients with SLE in Alexandria, Egypt. Alexandria is the second largest city after Cairo and the largest city on the Mediterranean coast. Second, we implemented random sampling in the selection of the participants; all participation was voluntary. Third, we deployed the 36-item Short Form Health Survey questionnaire (SF-36), which is a validated instrument to assess disease impact on HRQoL. We speculate that this study could increase the attention of the government and public health managers towards improving the quality of life of SLE patients.

### Supplementary Information

Supplementary Information.

## Data Availability

The datasets used and/or analyzed during the current study are not publicly available due to privacy and ethical concerns but are available from the corresponding author on reasonable request.

## Abbreviations

BP: Bodily pain
GH: General Health
HIPH: High Institute of Public Health
HRQoL: Health-related quality of life
IQR: Inter quartile range
MCS: Mental component score
MH: Mental Health
P: Probability
PCS: Physical component score
PF: Physical functioning
PRO: Patient-reported outcomes
RE: Role limitations in usual role activities because of emotional problems
ρ (Rho): Spearman rank-order correlation coefficient (non-parametric)
RP: Role limitations in usual role activities because of physical health problems
SD: Standard deviation
SF: Social functioning
SF-36: 36-Item short form health survey questionnaire
SLE: Systemic lupus erythematosus
SLICC: Systemic Lupus International Collaborating Clinics
SPSS: Statistical package for social sciences
U: The test statistic for the Mann Whitney non parametric Test
UV: Ultraviolet
VIT: Vitality
